# Synthesis of Iron Oxide/Gold Composite Nanoparticles Using Polyethyleneimine as a Polymeric Active Stabilizer for Development of a Dual Imaging Probe

**DOI:** 10.3390/nano8050300

**Published:** 2018-05-05

**Authors:** Gyu Jin Yoon, So Young Lee, Seung Bin Lee, Ga Young Park, Jin Hyun Choi

**Affiliations:** 1 Department of Advanced Organic Materials Science and Engineering, Kyungpook National University, Daegu 41566, Korea; creatdesign@naver.com (G.J.Y.); lsy9820@knu.ac.kr (S.Y.L.); windbin09@naver.com (S.B.L.); 2 Department of Bio-fibers and materials Science, Kyungpook National University, Daegu 41566, Korea; park_gayoung@knu.ac.kr

**Keywords:** iron oxide, gold, molecular imaging, composite nanoparticles, polyethyleneimine, polymeric active stabilizer, nanoseeds, alloy clusters

## Abstract

The combination of magnetic and plasmonic properties using iron oxide/gold nanocomposite particles is crucial for the development of multimodal molecular imaging probes. In this study, iron oxide/gold composite nanoparticles (NanoIOGs) were synthesized via the on-site reduction of an Au precursor salt by polyethyleneimine (PEI) molecules attached to iron oxide nanoparticles (IONPs), and they were employed in magnetic resonance and dark-field microscope imaging. PEI is considered as a polymeric active stabilizer (PAS), acting as a reducing agent for the synthesis of Au and a dispersant for nanoparticles. When the IONPs prepared at the PEI concentration of 0.02 wt. % were used for the NanoIOG synthesis, Au nanoseeds were formed around the IONPs. The alloy clusters of IONPs/Au crystals were produced with further reduction depending on PEI concentration. The NanoIOGs exhibited superparamagnetism in a magnetic field and plasmonic response in a dark-field (DF) microscope. The sizes, morphologies, magnetizations, and *r*_2_ relaxivities of NanoIOGs were affected significantly by the amount of PEI added during the NanoIOG synthesis. It is suggested that the PAS-mediated synthesis is simple and effective, and can be applied to various nanostructured Au-metal alloys.

## 1. Introduction

Ultrafine metal particles have received considerable attention because they offer promising and novel options for a wide range of applications. Nanoparticles with excellent colloidal stability have been synthesized for various applications because of their unique physical, chemical, and biological properties [1,2,3,4]. In particular, surface-modified superparamagnetic iron oxide nanoparticles (IONPs) have been widely used in novel biomedical applications, such as contrast enhancement in magnetic resonance (MR) imaging [5,6,7,8,9]. Polymer-coated gold nanoparticles (AuNPs) have been developed as potential X-ray computed tomography contrast agents owing to the high X-ray absorption coefficient of Au [10,11]. Furthermore, AuNPs with plasmonic properties are used in dark-field (DF) imaging [12,13] and surface-enhanced Raman spectroscopy [14]. Magnetic and plasmonic properties have been combined in iron oxide/gold composite nanoparticles (NanoIOGs) [15,16,17,18,19,20,21]. The poor spatial resolution of MR imaging can be overcome with DF microscopy because of the magnetoplasmonic effect of NanoIOGs as multimodal probes, which is particularly attractive for 3D imaging [20] or cell labeling [6,7,13].

To synthesize nanoparticles, metal ions must be reduced within a colloid, protecting against precipitation. Metal nanoparticles tend to agglomerate and form larger clusters, which is possibly overcome via the spontaneous adsorption of polymeric molecules onto the surface of particles [22]. In our previous study, ultra-small, polyethyleneimine (PEI)-capped IONPs were synthesized for MR imaging [23]. PEI is a cationic polymer with highly positive charges. The PEI adsorption layers on IONPs give a colloidal stability against agglomeration. Moreover, PEI molecules act as a stabilizer and reductant during the synthesis of AuNPs [24,25]. AuNPs can be produced in an aqueous PEI solution without a reducing agent such as NaBH_4_. Amino groups in PEI molecules function as proton acceptors/electron donors, reducing Au^3+^ to Au.

In most NanoIOG syntheses, chemical modifications, including ligand combination, are required for the selective formation of Au on the surfaces of IONPs. PEI has been used only as a dispersing agent in NanoIOG syntheses, and its reducing effect has been overlooked. We hypothesized that PEI molecules on the surfaces of IONPs could reduce Au ions, resulting in the on-site formation of Au crystals and eventually alloy clusters of iron oxide and Au. In this study, NanoIOGs were synthesized via the simple addition of an Au precursor salt to PEI-capped IONPs without chemical modification of IONP surfaces or addition of a reductant such as NaBH_4_. The effects of concentration of PEI on the synthesis of NanoIOGs were investigated. We also examined the magnetoplasmonic properties of NanoIOGs to verify their feasibility of application as MR/DF dual imaging probes.

## 2. Experimental Section

### 2.1. Synthesis of NanoIOGs

First, IONPs were synthesized via sonochemical co-precipitation of iron salts in aqueous PEI solutions with three different concentrations (0.02, 0.05, and 0.08 wt. % PEI). An aqueous solution containing 0.2 g of FeCl_3_·6H_2_O (>99%, Sigma-Aldrich, St. Louis, MO, USA) and 0.0825 g of FeCl_2_·4H_2_O (>99%, Sigma-Aldrich) was added to 100 mL PEI (branched, *M*_w_ ~ 25,000, Sigma-Aldrich) solution under magnetic stirring. After ultrasonic irradiation for 1 h, 1 mL of NH_4_OH solution (Duksan Pure Chemicals, Ansan, Korea) was added dropwise, and the mixture was stirred for 5 h at room temperature. The entire preparation procedure was conducted in N_2_ atmosphere. The resulting suspension was centrifuged at 15,000 rpm to remove large particles and the supernatant was dialyzed using a dialysis tube with a molecular weight cutoff of 1000 Da (Membrane Filtration Products Inc., Seguin, TX, USA). For the synthesis of Au, 3 mL of aqueous HAuCl_4_ solution (5.0 wt. %) was dropped into 100 mL of an aqueous solution of IONPs under magnetic stirring. The mixture was thereafter stirred for 3 h at room temperature to induce complete reduction, after which the solution was dialyzed for more than 3 h and thereafter centrifuged at 24,000 rpm. The precipitate was subsequently collected and dispersed in deionized water via ultrasonication to obtain the colloidal solution of NanoIOGs.

### 2.2. Characterization of NanoIOGs

To confirm the presence of Au and iron oxide crystals, X-ray diffraction (XRD) patterns of the NanoIOGs were obtained using an X-ray diffractometer (X’Pert PRO MRD, Philips, Amsterdam, The Netherlands) with Cu-Kα radiation. The NanoIOG colloidal solutions were treated with 65% nitric acid, and thereafter dispersed in a 3% nitric acid solution. The Fe concentration in each nanocolloid was determined using inductively coupled plasma-atomic emission spectrophotometry (Optima 7300DV, Perkin Elmer, Waltham, MA, USA). The morphology and composition of NanoIOGs were investigated using a field-emission scanning electron microscope (SEM) (SU8220, Hitachi, Tokyo, Japan) and a field-emission transmission electron microscope (TEM) (Titan G2 ChemiSTEM, FEI, The Netherlands) with an energy-dispersive X-ray (EDX) detector. Each NanoIOG colloidal solution was dropped onto a carbon-coated copper grid and dried at room temperature for 1 day for the preparation of a TEM sample. The mean hydrodynamic size of NanoIOGs was measured at room temperature using a ZEN 3600 dynamic light-scattering (DLS) instrument (Malvern Instruments, Malvern, UK). For TEM and DLS analyses, the NanoIOG colloidal solution with Fe concentration of 0.3 mM was used.

### 2.3. Magnetization and Relaxivity

To confirm superparamagnetic property, the magnetization of NanoIOGs was measured using a MPMS XL 7.0 superconducting quantum interference device magnetometer (Quantum Design, San Diego, CA, USA) at 300 K. The magnetic moment of each sample inserted into a gelatin capsule was measured in four quadrants under applied fields ranging from −50,000 to +50,000 Oe. A 9.4-T MR imaging scanner (BioSpec 94/20 USR, Bruker, Billerica, MA, USA), equipped with a 40 mm volume coil, was used to measure the *T*_2_ (transverse) relaxation times of aqueous NanoIOG solutions. The *r*_2_ proton relaxivity was determined from the slope of the plot of *R*_2_ relaxation rate (inverse of *T*_2_ relaxation time) versus Fe concentration. The typical measurement parameters were as follows: external MR field (*H*) = 9.4 T, temperature = 23 °C, number of acquisitions = 1, field of view = 34 mm × 34 mm, matrix size = 128 × 128, slice thickness = 1.0 mm, repetition time = 10,000 ms, and echo time = 10–1000 ms.

### 2.4. In Vitro Cytotoxicity and DF Microscopy

The NanoIOGs prepared with 0.08 wt. % PEI were suspended in a culture medium and used as a stock solution. Primary-cultured human fibroblast (HF) cells (1.0 × 10^6^), which were isolated from human skin provided by the Department of Plastics and Reconstructive Surgery in Kyungpook National University Hospital, Korea, were seeded with a growth medium (100 μL). These cells were treated with NanoIOGs by adding a fixed volume of the stock solution in order to dilute them to the required concentration, and thereafter incubated in a humidified atmosphere containing 5% CO_2_ at 37 °C for 48 h. The cells were subsequently washed twice with phosphate-buffered saline to remove any remaining particles. Subsequently, a fresh culture medium was added. After replacing the old medium, a 3-(4,5-dimethylthiazol-2-yl)-2,5-diphenyltetrazolium bromide (MTT) (Sigma-Aldrich) solution (5.0 gmL^−1^) was added to each well, and the cells were incubated for 4 h. The absorbance at 570 nm was measured using a microplate reader (Molecular Devices, San Jose, CA, USA). The cell viability (%) was expressed as the relative absorbance of the sample with respect to that of the control.

The bright field and DF microscope images of the HF cells cultured with the NanoIOGs (50 gmL^−1^ in a culture medium) were captured using an Axioplan 2 fluorescence microscope (Carl Zeiss, Oberkochen, Germany).

## 3. Results and Discussion

**Figure 1:**
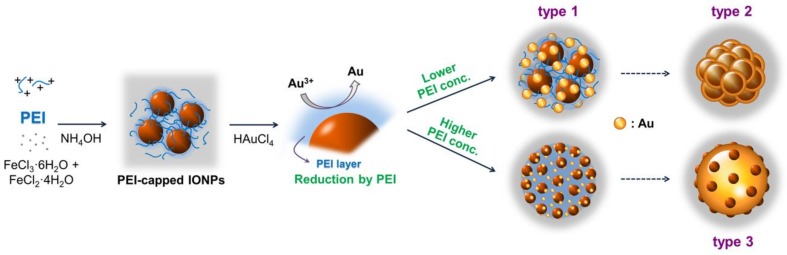
Schematic of the PEI-mediated NanoIOG synthesis.

The synthesis process of NanoIOGs containing iron oxide/Au alloy clusters is illustrated in Figure 1. First, the IONPs were prepared in the presence of PEI via the co-precipitation of Fe^2+^ and Fe^3+^. The PEI-capped IONPs were stable against oxidation and agglomeration. Subsequently, HAuCl_4_ was added to the colloidal dispersion of PEI-capped IONPs. During the chemical synthesis of gold, a reducing agent donates electrons, converting Au ions to metallic form. PEI molecules, containing a large number of amino groups in the long molecular chain, form complexes with Au ions via coordination and reduce them by donating electrons. The PEI molecules on IONP surfaces reduced Au ions, resulting in the on-site formation of Au crystals. The NanoIOGs containing iron oxide/Au alloy clusters were prepared via the direct addition of HAuCl_4_ to the stable colloidal solution of PEI-capped IONPs, without any other reducing agent.

**Figure 2:**
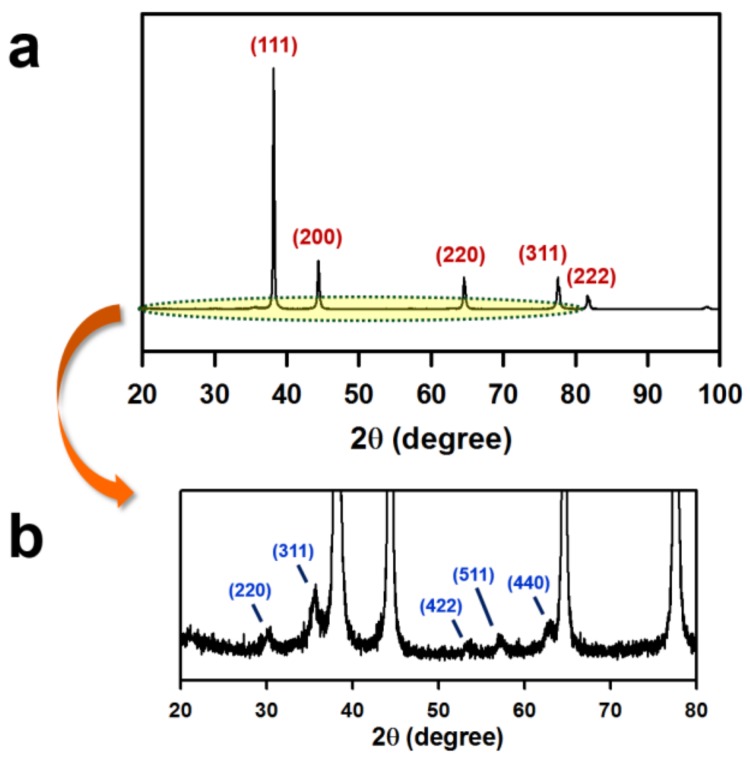
XRD patterns of the NanoIOGs for Au (**a**) and iron oxide (**b**) crystals. The diffraction patterns were almost identical for the NanoIOGs prepared with the PEI concentrations of 0.02, 0.05, and 0.08 wt. %.

In the XRD patterns (Figure 2), the peaks at 30°, 36°, 53°, 57°, and 63° are assigned to the reflections from the (220), (311), (422), (511), and (440) planes, respectively, of the iron oxide crystal. More distinct diffraction peaks are observed for the (111), (200), (220), and (311) planes of the face-centered-cubic Au crystals. The XRD analysis confirms the coexistence of iron oxide and Au crystals.

**Figure 3:**
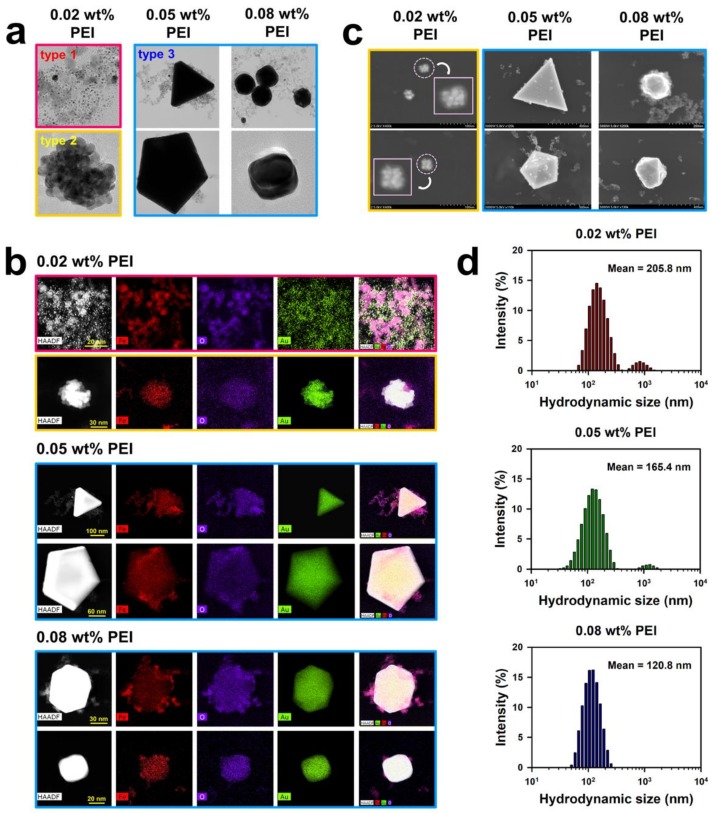
TEM images (**a**), high-angle annular dark-field (HAADF) scanning TEM images with the corresponding EDX elemental mappings for Fe, O, and Au (**b**), SEM images (**c**), and hydrodynamic sizes (**d**) of the NanoIOGs produced at different PEI concentrations.

Through the entire procedure of NanoIOG synthesis, PEI was used as a polymeric active stabilizer (PAS), acting as a reductant for the synthesis of gold and a dispersant for nanoparticles and nanoclusters. Thus, the synthesis of NanoIOGs was significantly influenced by the concentration of PEI. When the IONPs prepared at the PEI concentration of 0.02 wt. % were used, seed-like AuNPs were produced around the IONPs. The resulting product is a mixture of IONPs and Au nanoseeds (type 1), as shown in the TEM images and EDX elemental mappings in Figure 3. The Au nanoseed formation around the IONPs is the key point in elucidating the production of alloy clusters of IONPs/Au crystals in this study. The AuNP seeds were produced adjacent to the IONPs, because most of Au ions could be reduced by the adsorption layers of PEI on the IONPs. The cloud-like aggregates of Au nanoseeds and IONPs mixture were found in the TEM images of type 1 (also shown in Figure S1), and they can be regarded as precursors of the alloy clusters of iron oxide and gold. Based on the nanoseed-assisted synthesis of Au crystals around the IONPs, the alloy clusters could be produced depending on PEI concentration. The aggregated alloy clusters of Au-coated IONPs (type 2 in Figure 1) were also found at 0.02 wt. % PEI. The coexistence of iron oxide and Au in a single cluster was confirmed via EDX mapping images for type 2 (Figure 3b), which had a core (IONPs)-shell (Au) structure, as shown in the SEM images (Figure 3c). With further reduction by PEI, the Au seeds grew to form Au crystal layers on the surfaces of IONPs, resulting in the core-shell alloy clusters. However, the growth of Au crystals was limited because of the relatively low concentration of PEI as a reducing agent. When the IONPs prepared at the higher PEI concentrations, such as 0.05 and 0.08 wt. %, were used, larger alloy clusters (type 3) were produced, as shown in Figure 1 and Figure 3b. Under these conditions, the nucleation and growth of Au seeds proceeded intensively via vigorous reduction by PEI. The iron oxide/Au alloy clusters produced with 0.05 wt. % PEI were mostly large and well-faceted, whereas those formed with 0.08 wt. % PEI were relatively small and nearly spheroidal, as shown in Figure 3a,c, indicating the dispersing effect of PEI on the size and shape of alloy cluster.

In our previous study, the particle and hydrodynamic cluster sizes of IONPs were strongly influenced by the concentration of PEI [23]. Higher concentrations of PEI enhanced the effectiveness of the polymer as a stabilizer, lowering the particle and cluster sizes. This phenomenon may also apply to the NanoIOG synthesis in this study. In the first step of NanoIOG synthesis, PEI was used as a capping and dispersing agent for IONPs. It is apparent that the amount of PEI affected the sizes of IONPs and their clusters. In the second step, PEI acted as a reducing agent for Au and a dispersant for the NanoIOGs. The complicated interactions associated with the use of PEI as a PAS may be responsible for the effect of concentration on the size and morphology of NanoIOGs. In Figure 3d, the hydrodynamic sizes of NanoIOGs produced at different PEI concentrations are shown. Although the particles sizes of Au seeds and iron oxide/Au alloy clusters were mostly small (below 100 nm), the mean hydrodynamic size of NanoIOGs produced with 0.02 wt. % PEI was 205.8 nm, owing to poor stabilization and high degree of aggregation. Under a perfect stabilizing condition, each nanoparticle should be isolated without aggregation, so that the hydrodynamic size measured by DLS should be almost the same value with the individual particle size. Unfortunately, the dispersing effect was weak when the 0.02 wt. % PEI was used, so that the mixture of Au seeds and IONPs formed cloud-like aggregates of type 1. The hydrodynamic size of type 1 aggregates and type 2 clusters should be measured by DLS in this case. For this reason, the size distribution of individual Au nanoseeds were not displayed, as shown in Figure 3d, suggesting that there were neither a single Au seed particle nor its small aggregate, which can have an exclusive volume to be measured as a hydrodynamic size due to aggregation. Instead, we measured the size of individual Au seeds from TEM images and provided the corresponding diagram in Figure S2. The average size of the Au seeds was calculated at 2 nm. At 0.05 wt. % PEI, large iron oxide/Au alloy clusters were formed and possibly aggregated with each other. More PEI molecules could be required for better dispersion of the large clusters. Therefore, the NanoIOGs produced with this concentration of PEI had a larger hydrodynamic size than those produced at 0.08 wt. % PEI. The size of iron oxide/Au alloy clusters was relatively small, and the aggregation of particles was well-screened, resulting in a lower hydrodynamic size of the NanoIOGs produced at 0.08 wt. % PEI.

**Figure 4:**
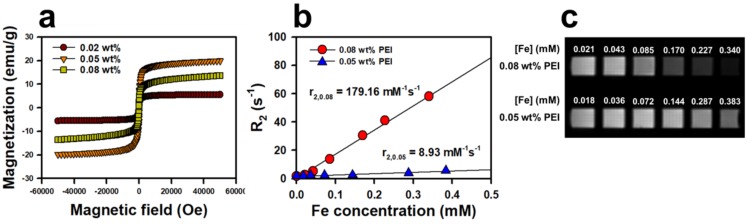
Magnetization curves for the NanoIOGs produced with different PEI concentrations; the inset shows an enlarged plot measured with magnetic field strengths between −2000 and +2000 Oe (**a**), *R*_2_ relaxation rate versus Fe concentration for the NanoIOGs prepared with 0.05 and 0.08 wt. % PEI; The slope from the linear regression yields the *r*_2_ relaxivity (**b**), and *T*_2_-weighted MR images (echo time = 0.03 s) (**c**).

The superparamagnetic properties of IONPs are attributed to the combination of high magnetization of ferromagnetic bulk iron oxide and the paramagnetic nature of iron ions. The characteristics of this superparamagnetism are a large magnetic moment in the presence of an externally applied magnetic field and the absence of a remnant magnetic moment when this applied field is reduced to zero. In Figure 4a, the net magnetization returns almost to zero in the hysteresis loops of the magnetization curves, which indicates that the NanoIOGs are superparamagnetic. The saturation-magnetization (*M*_s_) values for NanoIOGs prepared with 0.02, 0.05, and 0.08 wt. % PEI were 5.6, 19.9, and 13.6 emu/g, respectively. It has been reported that *M*_s_ and the size of IONPs are correlated linearly [22,26,27]. As the IONPs grow, their surface-to-volume ratios decrease. This reduces surface effects such as unsatisfied bonds, non-collinear spins, spin canting, and spin-glass-like behavior, which can increase *M*_s_. It is inferred that the presence of iron oxide/Au alloy clusters produced with 0.05 and 0.08 wt. % PEI contributed to the enhancement of *M*_s_, as they can behave as large iron oxide crystals in a magnetic field. However, the NanoIOGs synthesized with 0.02 wt. % PEI had difficulty forming large clusters, and thus exhibited the lowest *M*_s_ values. Owing to the surface modification of IONPs, a magnetically inactive layer is formed, which reduces *M*_s_ by creating an asymmetric atomic environment on the surface [28,29]. The thicknesses of PEI layers on the surfaces of NanoIOGs can increase with the concentration of PEI. The NanoIOGs prepared with 0.05 wt. % PEI were larger, with fewer PEI molecules on their surfaces, resulting in the enhancement of *M*_s_ as compared with those prepared with 0.08 wt. % PEI.

Superparamagnetic IONPs enhance the proton spin–spin relaxation and thus reduce the *T*_2_ relaxation time. The ability of IONPs to exhibit MR imaging contrast is related to the net effectiveness of the reduction of relaxation time, which is known as *r*_2_ relaxivity. As shown in Figure 4b, the *r*_2_ relaxivity was evaluated from the change in *R*_2_ relaxation rate per unit concentration of the NanoIOGs prepared with 0.05 and 0.08 wt. % PEI. For those prepared with 0.02 wt. % PEI, no linear relationship between *R*_2_ and concentration was obtained because the resulting NanoIOG solution exhibited poor dispersion. Remarkably, *r*_2_ of NanoIOGs prepared with 0.08 wt. % PEI was 179.2 mM^−1^ s^−1^ and was much higher than that (29.5 mM^−1^ s^−1^) of IONPs prepared with the same PEI concentration [25]. This suggests that the presence of iron/Au alloy clusters increases the *r*_2_ relaxivity. If severe aggregation does not occur, *r*_2_ of IONPs should increase with the particle and cluster sizes in the same manner as *M*_s_ [30]. The presence of well-dispersed iron oxide/Au alloy clusters increased the average iron oxide cluster size and *r*_2_ relaxivity. In addition, the magnetically inactive PEI layers could decline as Au crystals were formed on the surfaces of IONPs, resulting in the enhancement of *r*_2_ relaxivity as compared with the IONPs produced with the same PEI concentration. On the contrary, the reverse is observed for IONP aggregates larger than 200 nm. A very strong magnetic field is generated by these aggregates, and thus, the nearby water protons are completely dephased and cannot contribute to the MR signal [30]. In the case of 0.05 wt. % PEI, the *r*_2_ relaxivity of IONPs was 227.6 mM^−1^ s^−1^ in our previous study [25]. However, the *r*_2_ of NanoIOGs produced with the same PEI concentration decreased significantly to 8.9 mM^−1^ s^−1^ because the NanoIOGs contained clusters larger than 200 nm. These indicate that the stabilizing effect of PEI and the size control of NanoIOGs are very important for their application as MR imaging probes. Reduction of particle size is necessary to prepare a more stable colloidal dispersion of NanoIOGs. Addition of more PEI (higher than the 0.08 wt. %) or other stabilizing agent could be valid in order to reduce particles size; however, the more magnetically inactive stabilizer molecules may result in the lower *M*_s_ and *r*_2_. In our previous study, the IONPs prepared at 0.05 wt. % PEI revealed higher *M*_s_ and *r*_2_ than those prepared at 0.08 wt. % PEI, for this reason. Hence, the optimization of PAS concentration in NanoIOGs synthesis is very important.

**Figure 5:**
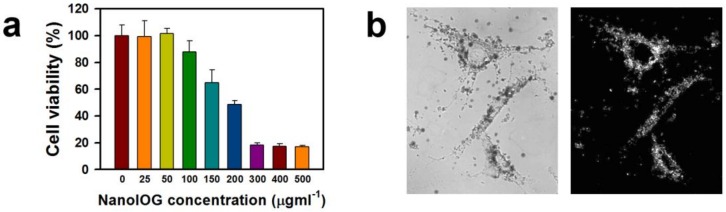
Relative viability of human fibroblast cells cultured with the NanoIOGs prepared with 0.08 wt. % PEI; the error bars indicate ±SD (*n* = 4) (**a**), and bright field (left) and DF (right) microscope images of human fibroblast cells cultured with the NanoIOGs prepared with the same PEI concentration (**b**).

The cytotoxicity of NanoIOGs prepared with 0.08 wt. % PEI was estimated using an MTT assay. The cytotoxic effects of bare IONPs are well known. They induce a loss of 20% in the viability of human fibroblasts at 50 ppm [31]. The cytotoxicity of IONPs has been linked to cellular uptake followed by the production of reactive oxygen species. Nevertheless, it is clear that in the clinical dosages typically used for imaging, the amount of IONPs injected to obtain good contrast is not sufficiently high to be toxic to patients or animals [27]. On the other hand, AuNPs are regarded as biocompatible, and acute cytotoxicity has not been observed [32]. The presence of IONPs is probably responsible for the cytotoxicity of NanoIOGs. In addition, the strong electrostatic interactions between the positively charged polymer backbones and negatively charged cell membranes are responsible for the cytotoxicity of PEI [33]. The NanoIOGs caused no cytotoxicity to human fibroblasts at 50 ppm and resulted in a reduction of 12% in cell viability at 100 ppm (Figure 5a), indicating that the Au coating on the IONPs may reduce the cytotoxicity. The cytotoxic effect of PEI and IONPs could be inhibited due to the Au crystal formation on the surfaces of PEI-capped IONPs.

The plasmonic and light scattering properties of AuNPs enable DF imaging. The optical image of human fibroblast cells treated with NanoIOGs was well-demonstrated via DF microscopy as shown in Figure 5b. The NanoIOGs are brightly illuminated in the DF, whereas the IONPs do not scatter light. The AuNPs may require enhanced aggregation and accumulation for clear DF microscopy visualization, whereas the NanoIOGs contain large Au clusters and thus offer advantages for rapid DF imaging of live cells.

## 4. Conclusions

We synthesized NanoIOGs via the on-site reduction of an Au precursor salt with PEI molecules attached to IONPs. The PEI-capped IONPs were prepared with different PEI concentrations, and HAuCl_4_ was added to the PEI-IONP colloidal dispersions without chemical modification of the surfaces of IONPs and addition of any other reductants. When the reduction was mild at the relatively low concentration of PEI, Au nanoseeds were formed around the IONPs. With further reduction by PEI, the Au seeds grew to form Au crystal layers on the surfaces of IONPs, resulting in the core-shell alloy clusters. When the IONPs prepared at the higher PEI concentrations were used, the larger alloy clusters were produced The NanoIOGs, which contained the alloy clusters of IONPs/Au crystals, exhibited magnetoplasmonic effects useful in MR and DF imaging. PEI played a key role in the NanoIOG synthesis as a dispersing and reducing agent, and hence, the sizes, morphologies, magnetizations, and *r*_2_ relaxivities of NanoIOGs were influenced significantly by the concentration of PEI. The presence of well-dispersed iron/Au alloy clusters enhanced the *r*_2_ relaxivity remarkably. It is suggested that the PAS-mediated synthesis is an effective method for synthesizing Au–metal alloy nanoparticles.

## Supplementary Materials

The following are available online at http://www.mdpi.com/2079-4991/8/5/300/s1, Figure S1: EDX elemental mapping-merged HAADF scanning TEM images of the type 1 NanoIOGs produced at 0.02 wt. % PEI; Figure S2: Size distribution for the Au nanoseeds produced at 0.02 wt. % PEI measured from TEM images (*n* = 200).
