# Supplementary material for: Synthesis of Iron Oxide/Gold Composite Nanoparticles Using Polyethyleneimine as a Polymeric Active Stabilizer for Development of a Dual Imaging Probe
Source: Nanomaterials (Basel). 2018 May 5;8(5):300. doi: 10.3390/nano8050300 (PMC5977314; doi:10.3390/nano8050300)
Supplement: Supplementary file 1 [file nanomaterials-08-00300-s001.pdf]

Supplementary Materials

.

Synthesis of Iron Oxide/Gold Composite Nanoparticles Using Polyethyleneimine as a Polymeric Active Stabilizer for Development of a Dual Imaging Probe

Gyu Jin Yoon, So Young Lee, Seung Bin Lee, Ga Young Park and Jin Hyun Choi

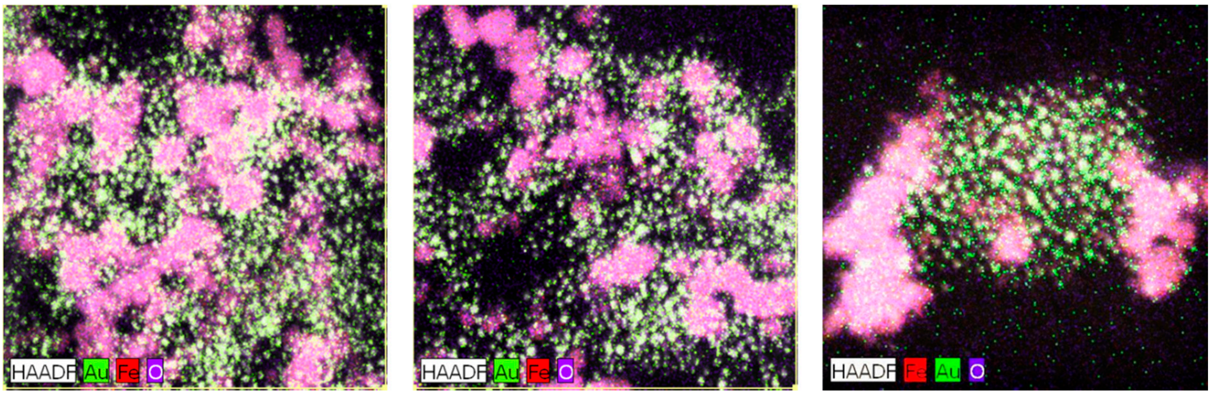

Figure S1: EDX elemental mapping-merged HAADF scanning TEM images of the type 1 NanoIOGs produced at 0.02 wt. % PEI.

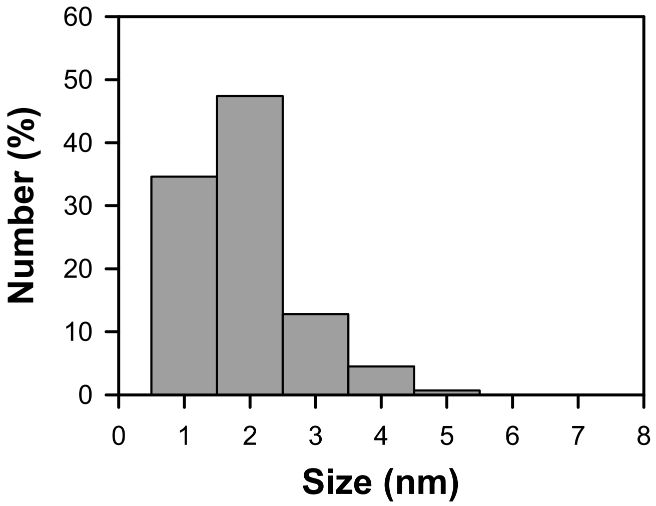

Figure S2: Size distribution for the Au nanoseeds produced at 0.02 wt. % PEI measured from TEM images (n = 200).
